# Supplementary material for: Vaccination card loss and associated factors in Ethiopia: A multilevel analysis using Ethiopia Mini Demographic and Health Survey 2019 data
Source: PLoS One. 2024 Dec 18;19(12):e0311925. doi: 10.1371/journal.pone.0311925 (PMC11654936; doi:10.1371/journal.pone.0311925)
Supplement: Attachment — Submitted filename: Response to reviewers.docx [file pone.0311925.s001.docx]

PONE-D-24-15363

Vaccination Card Loss and Associated Factors in Ethiopia: A Multilevel Analysis using Ethiopia Mini Demographic and Health Survey 2019 Data

PLOS ONE
Dear Dr. Erchafo,
Thank you for submitting your manuscript to PLOS ONE. After careful consideration, we feel that it has merit but does not fully meet PLOS ONE’s publication criteria as it currently stands. Therefore, we invite you to submit a revised version of the manuscript that addresses the points raised during the review process.

Please review and address the reviewer's comment .
Please submit your revised manuscript by Sep 21 2024 11:59PM. If you will need more time than this to complete your revisions, please reply to this message or contact the journal office at [plosone@plos.org](mailto:plosone@plos.org). When you're ready to submit your revision, log on to <https://www.editorialmanager.com/pone/> and select the 'Submissions Needing Revision' folder to locate your manuscript file.
Please include the following items when submitting your revised manuscript:

- A rebuttal letter that responds to each point raised by the academic editor and reviewer(s). You should upload this letter as a separate file labeled 'Response to Reviewers'.
- A marked-up copy of your manuscript that highlights changes made to the original version. You should upload this as a separate file labeled 'Revised Manuscript with Track Changes'.
- An unmarked version of your revised paper without tracked changes. You should upload this as a separate file labeled 'Manuscript'.

If you would like to make changes to your financial disclosure, please include your updated statement in your cover letter. Guidelines for resubmitting your figure files are available below the reviewer comments at the end of this letter.
If applicable, we recommend that you deposit your laboratory protocols in [protocols.io](http://protocols.io/) to enhance the reproducibility of your results. Protocols.io assigns your protocol its own identifier (DOI) so that it can be cited independently in the future. For instructions see: <https://journals.plos.org/plosone/s/submission-guidelines#loc-laboratory-protocols>. Additionally, PLOS ONE offers an option for publishing peer-reviewed Lab Protocol articles, which describe protocols hosted on [protocols.io](http://protocols.io/). Read more information on sharing protocols at <https://plos.org/protocols?utm_medium=editorial-email&utm_source=authorletters&utm_campaign=protocols>.

We look forward to receiving your revised manuscript.

Kind regards,

Dipendra Khatiwada, MD
Academic Editor
PLOS ONE

**Dear Academic Editor and Reviewers:**

Thank you for providing us an opportunity to revise and resubmit our paper. Below we address all the questions and concerns raised by the academic editor and reviewers in a point-by-point fashion.

Journal Requirements:

When submitting your revision, we need you to address these additional requirements.

1. Please ensure that your manuscript meets PLOS ONE's style requirements, including those for file naming. The PLOS ONE style templates can be found at <https://journals.plos.org/plosone/s/file?id=wjVg/PLOSOne_formatting_sample_main_body.pdf> and 
<https://journals.plos.org/plosone/s/file?id=ba62/PLOSOne_formatting_sample_title_authors_affiliations.pdf>

Response: the revised manuscript follows the PLOS ONE format.

2. We note that your Data Availability Statement is currently as follows: [All relevant data are within the manuscript and its Supporting Information files.]

Please confirm at this time whether or not your submission contains all raw data required to replicate the results of your study. Authors must share the “minimal data set” for their submission. PLOS defines the minimal data set to consist of the data required to replicate all study findings reported in the article, as well as related metadata and methods (<https://journals.plos.org/plosone/s/data-availability#loc-minimal-data-set-definition>).

For example, authors should submit the following data:

- The values behind the means, standard deviations and other measures reported;
- The values used to build graphs;
- The points extracted from images for analysis.

Authors do not need to submit their entire data set if only a portion of the data was used in the reported study.

If your submission does not contain these data, please either upload them as Supporting Information files or deposit them to a stable, public repository and provide us with the relevant URLs, DOIs, or accession numbers. For a list of recommended repositories, please see <https://journals.plos.org/plosone/s/recommended-repositories>.

If there are ethical or legal restrictions on sharing a de-identified data set, please explain them in detail (e.g., data contain potentially sensitive information, data are owned by a third-party organization, etc.) and who has imposed them (e.g., an ethics committee). Please also provide contact information for a data access committee, ethics committee, or other institutional body to which data requests may be sent. If data are owned by a third party, please indicate how others may request data access.

Response: The original data set of 2019 EMDHS is available from EDHs program on request using the following Link (<https://dhsprogram.com/publications/index.cfm>)

3. Please review your reference list to ensure that it is complete and correct. If you have cited papers that have been retracted, please include the rationale for doing so in the manuscript text, or remove these references and replace them with relevant current references. Any changes to the reference list should be mentioned in the rebuttal letter that accompanies your revised manuscript. If you need to cite a retracted article, indicate the article’s retracted status in the References list and also include a citation and full reference for the retraction notice.

Response: we appreciate the comment and we have incorporated the updated references in the revised copy.

Reviewer #1: Vaccination card retention is a very interesting and under-looked researched topic. The researchers have tried to bring about factors associated with it.

My comments are:

Ln 52 - 54: (Recommendation in abstract): HCP should be encouraged to provide vaccination cards to mothers or caregivers of children.
-> This is not based on the findings of the study.

Response: we appreciate the comment and we have incorporated it in the revised copy.
StatisticalAnalysis:
Ln 164: .. applied the multilevel logistic regression model with region-specific random effects..
-> Need to further clarify if it is - fixed effect random intercept, or random effect random intercept model.

Response: random effect random intercept model.
Ln 167 - 177: Details of model M3 is required. It is not clear how the model was formed.

Response: In M3, since we have only one independent variable at regional level (Residence), we didn’t apply variable selection technique to determine the covariates to be included in this model. Because of that we didn’t discuss about m3. However, it is candidate for M4. So, now we have incorporated it in the revised copy.

Result

Ln 204, Ln 230, Ln 262: The mean plus standard deviation ...
-> The SD has been kept in parenthesis rather than after plus sign. So, mention accordingly.
 Response: we appreciate the comment and we have incorporated it in the revised copy.

Ln 258, Ln 292: Table 2,3: Do mention the notation Mean(SD) or N(%) in the table as well.

Response: we appreciate the comment and we have incorporated it in the revised copy.

Figure 3: caption: presence of vaccination ...
-> Keep in sentence case (Presence of ..)

Response: we appreciate the comment and we have incorporated it in the revised copy.

Ln 333: ... with 31.5% of the variance among observations being attributed
-> Complete the sentence.

Response: we appreciate the comment and we have incorporated it in the revised copy.

Ln 337: Table 4 caption does not mention about M3 model.

Response: As we have mentioned above, M3 was applied at reginal level and had only one independent variable. So, M3 was not candidate for model selection.

Discussion:
Ln 412: ..education has a positive association with vaccination card loss.
-> the association can be better called negative, as with increase education, there is reduced vaccination card loss.

Response: we have addressed it in the revised copy as follows:

In this study, respondents’ with low education status are more likely to experience child vaccination card loss.

Ln 421: ... wealth index had a positive association..
-> we can call it positive association with vaccination card retention rather than the card loss.

Response: we have addressed it in the revised copy as follows:

In this study, respondents with the poorest wealth index status are more likely to experience the loss of their child's vaccination card.

Ln 432: The age of respondents was another determinant factor for vaccination card loss.
-> The table 5 reports age of HH, while age of respondent is mentioned here.
-> And, the contrasting findings should be discussed, with plausible reasons for the finding of decrease in vaccination card loss with increasing age- reported in this study.

Response: we have addressed it in the revised copy as follows:

As the age of respondents increases, there is the possibility of repeated experiences on the importance of having vaccination cards of their children for different events, like as prerequisite evidence for joining the schools and for certificates with regard to vital events in the country. These all experiences enhance vaccination card retention as age increases.

Ln 433-434: A study in Cameron. Contrasts this finding...
-> Remove the period and correct the sentence.

Response: we appreciate the comment and we have incorporated it in the revised copy.
Ln 437-438: the findings of this study is contrary to the study done in Nepal Nepal where higher vaccination card retention ....
-> Reframe and correct the sentence grammatically.

Response: we appreciate the comment and we have incorporated it in the revised copy.
Ln 481- 483: .. recording birth interval, mothers who gave ..
-> This finding also requires discussion.

Response: we have addressed it in the revised copy as follows:

Having an additional child in a short birth interval and accompanying the other child make the mother busy and forget about safekeeping of the vaccination card.

Limitation - seems to be missing (if there is any in the study). The limitations can be regarding the model selection or others if any.

Response: we have addressed it in the revised copy as follows:

The limitation of this study is investigating the regional-level variation without considering the district-level.

Reviewer #2: The study's sampling design and methods could have been more detailed to eliminate any biases.

Response: we appreciate the comment, and we used the EDHS sampling design and methods, and the data source was also EDHS.

Details on how the random effects were specified and the rationale for choosing specific levels would enhance transparency.

Response: we appreciate the comment and we have incorporated it.
The methodology could give a detailed explanation of how potential confounders were controlled and whether there was multicollinearity among variables.

Response: we appreciate the comment and we have incorporated it.
